# Clinical practice patterns for radiotherapy in older patients with head and neck cancer: Results of the ESTRO head and neck focus group patterns-of-care survey

**DOI:** 10.1016/j.ctro.2026.101351

**Published:** 2026-11-04

**Authors:** Erik Haehl, Alexander Rühle, Panagiotis Balermpas, Jon Cacicedo, Franziska Walter, Claus Belka, Nils Henrik Nicolay

**Affiliations:** a Department of Radiation Oncology, LMU University Hospital, LMU Medizin, Ludwig-Maximilians-Universität München, Marchioninistr. 15, 81377 Munich, Germany; b Bavarian Cancer Research Center (BZKF), Munich, Germany; c Department of Radiation Oncology, University of Leipzig Medical Center, Stephanstraße 9a, 04103 Leipzig, Germany; d Comprehensive Cancer Center Central Germany, Partner Site Leipzig, Leipzig, Germany; e Department of Radiation Oncology, University Hospital Zurich (USZ), University of Zurich (UZH), Rämistrasse 100, CH-8091 Zurich, Switzerland; f Department of Radiation Oncology, Cruces University Hospital/Biobizkaia Health Research Institute, Barakaldo, Spain

**Keywords:** Head and neck cancer, HNSCC, Elderly, Older patients, ESTRO Head and Neck Focus Group, Patterns-of-care

## Abstract

**Introduction:**

Demographic projections predict a steadily growing number of older patients with head and neck squamous cell carcinoma (HNSCC). As they remain underrepresented in clinical trials, substantial uncertainty persists in their radiotherapeutic management. We aimed to map current international practice and identify areas of heterogeneity that warrant evidence-generating efforts and consensus development.

**Methods:**

On behalf of the Head and Neck Focus Group of the European Society for Radiotherapy and Oncology (ESTRO), we conducted an international web-based survey covering 34 questions on pretreatment work-up, treatment parameters and supportive care. The survey was distributed via the ESTRO mailing list and social media between September and December 2025.

**Results:**

85 radiation oncologists responded, predominantly from academic institutions. Of those providing geographic origin (*n* = 63) 41% are practicing outside Europe. Age thresholds defining older HNSCC patients ranged from 65 to 75 years and were significantly lower among non-European respondents (*p* < 0.001), whereas structured comorbidity scoring (32%) and geriatric screening (25%) were applied by a minority. PET/CT was used by 62% of respondents for pretreatment imaging. Bilateral elective nodal irradiation was commonly used in the definitive (86%) but less often in the adjuvant setting (66%). Around half of respondents routinely omitted chemotherapy beyond a self-defined age threshold. Weekly cisplatin was the most frequent regimen for both definitive (60%) and adjuvant (63%) chemoradiation treatments. Hypofractionation was the most frequently reported alternative in case of chemotherapy ineligibility (41%).

**Conclusion:**

This international survey on the treatment of older patients with HNSCC identifies areas of relative consensus among responding radiation oncologists as well as clinically relevant heterogeneity in age-based chemotherapy decisions, geriatric assessment, postoperative target volumes, and supportive care.

## 1 Introduction

Head and neck squamous cell carcinoma (HNSCC) is the seventh most common malignancy worldwide [1]. In Western populations, approximately one quarter of HNSCC patients are aged 70 years or older at diagnosis [2], and demographic projections indicate a growing number of older HNSCC patients requiring oncological treatment [3].

Treatment standards for locoregionally advanced HNSCC comprise definitive chemoradiation or surgery followed by risk-adapted (chemo-)radiation [4]. However, the landmark trials either excluded or underrepresented older patients [5], [6], [7], [8]. When older patients were specifically addressed in clinical studies, the survival benefit of treatment intensification such as concomitant chemotherapy or altered fractionation declined with advancing age [9], [10]. Consequently, evidence-based treatment recommendations for older HNSCC patients remain limited and have largely been extrapolated from younger cohorts.

Older HNSCC patients differ from younger patients beyond chronological age. Age-related comorbidity is independently prognostic for survival [11], [12] and moderates the benefit of concomitant systemic therapy [13]. Yet comorbidity captures only part of the age-related vulnerability. Frailty, a multidimensional functional construct largely independent of chronological age, predicts both outcomes and treatment feasibility [14], [15]. This is particularly relevant in HNSCC, where curative treatment carries considerable toxicity to which older patients appear especially vulnerable. Radiotherapy alone is generally feasible [16], although higher rates of acute mucositis and functional toxicity were reported in older patients [17], [18]. Evidence is more consistent for chemotherapy toxicity, particularly cisplatin-induced nephrotoxicity, ototoxicity and myelosuppression [19]. These risks have to be weighed against differing treatment goal prioritization of older patients: while cure remains the primary objective irrespective of age, preservation of quality of life and overall treatment tolerability play a particularly important role in this population [20].

Recent prospective and registry data increasingly inform the management of older HNSCC patients. The international SENIOR cohort demonstrated a survival benefit for concomitant chemotherapy in older patients that decreased with advancing age [13], [21]. The ELAN-RT trial investigated a hypofractionated split-course regimen for frail older patients with high locoregional control and lower acute toxicity [22]. Despite this emerging evidence, prior pattern-of-care surveys consistently reported substantial heterogeneity in the management of older HNSCC patients, including the use of geriatric screening, cisplatin eligibility criteria, fractionation, and supportive interventions [23], [24], [25], [26]. These previous surveys, however, were either regional in scope or conducted before the publication of the most recent evidence base.

We therefore initiated a survey through the European Society for Radiotherapy and Oncology (ESTRO) Head and Neck Focus Group addressing pretreatment work-up, indications and choice of concomitant systemic therapy, fractionation regimens, and supportive interventions. The aim of this study was to map current European and international practice in the radiation oncology management of older HNSCC patients to identify areas of heterogeneity and to inform future therapy refinements and consensus initiatives.

## 2 Methods

This study was an international, web-based survey initiated through the ESTRO Head and Neck Focus Group. Design, conduct, and reporting were informed by the ESTRO guidelines for survey-based radiation oncology research.

The questionnaire comprised 34 questions: 4 on pretreatment procedures, 14 on treatment parameters, 5 on supportive care, 2 example cases with 7 case-specific questions and 4 questions on supplier information. Questions were multiple-choice with single or multiple response options. Individual questions could be skipped; no skip logic or forced-response setting was applied. All percentages refer to the number of respondents answering the respective question. The full questionnaire is provided in supplementary material. Guideline examples were named in the respective response options asking for guideline adherence. (**Supplementary Table 9**).

The questionnaire was developed by three of the authors (EH, AR, NHN), all radiation oncologists with a research focus on older HNSCC patients, and was informed by the experience gained in a previously published trinational survey of German-speaking countries [23], from which the core items were adapted and extended for an international audience. The draft was reviewed and formally approved by all 18 members of the ESTRO Head and Neck Focus Group. No separate pilot test or cognitive testing was performed. The questionnaire was available in English only.

The survey was open from September 23 to December 19, 2025, distributed via the ESTRO mailing list and the official ESTRO social media channels. Responses were collected using the ESTRO SurveyMonkey account (SurveyMonkey, San Mateo, CA, USA). Participation was voluntary, and responses were collected anonymously. The distributed link was open and non-personalized and no respondent-identifying data were collected. The number of individuals who received or viewed the invitation could therefore not be determined and repeated participation of the same individual could not be technically excluded, precluding conventional response rate calculation.

Descriptive statistics summarized responses. Comparisons between European and non-European respondents, classified by country of practice as listed in Supplementary Table 10, were performed post hoc and were the only inferential analyses conducted. As expected cell counts below five occurred in both comparisons, groups were compared by Fisher's exact test without collapsing response categories; Pearson's chi-squared results are provided in Supplementary Tables 2 and 3. Cramér's V is given as effect size. Two-sided *p*-values <0.05 were considered significant; no correction for multiple testing was applied, and all inferential results are exploratory.

Given the anonymous design and absence of patient-related data, formal ethics approval was not required.

## 3 Results

Eighty-five radiation oncologists answered the questionnaire, of whom 63 (74.1%) reached the final questionnaire section. Detailed response rates to all questions are provided in the supplementary material. 63 respondents stated their geographic origin. Of those, most respondents were practicing in Europe (*n* = 37, 58.7%), followed by Asia (*n* = 20, 31.7%) and North America (*n* = 4, 6.3%), Africa (*n* = 1, 1.6%) and Australia (*n* = 1, 1.6%). Within Europe, most practiced in Western Europe (45.9%), followed by equal proportions from Northern (18.9%), Eastern (18.9%) and Southern Europe (16.2%). Professional expertise was generally high with 96.9% of board-certified radiation oncologists and 76.6% working in academic centers. 45.3% stated ten or more years of experience and 50.0% reported treating more than 50 older HNSCC patients annually (Fig. 1**, Supplementary Table 1 and 10**).

**Fig. 1:**
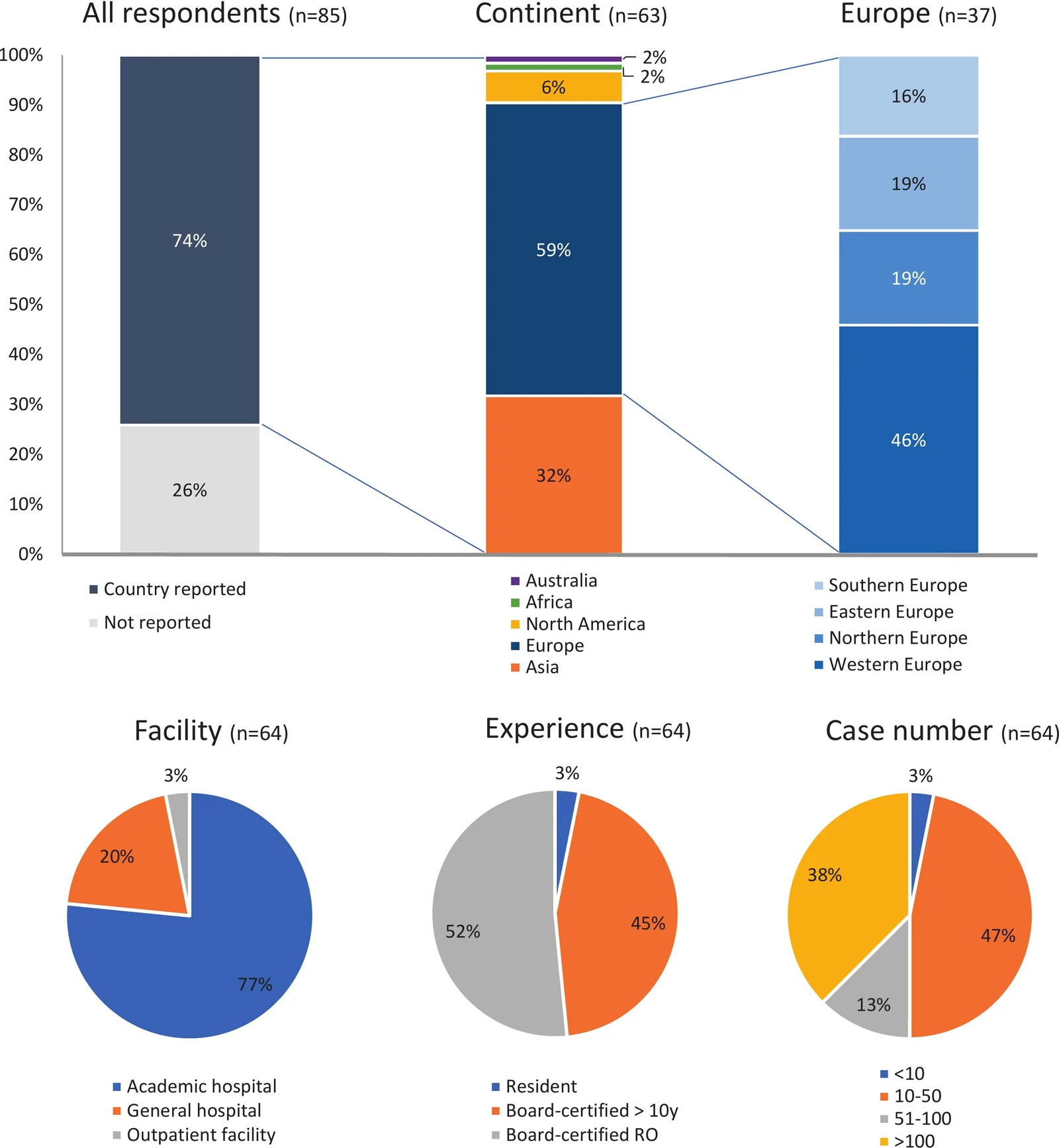
Stacked bar charts of respondents' geographic origin: availability of country information among all respondents (*n* = 85) with distribution by continent (*n* = 63) and breakdown of European sub-regions (*n* = 37); pie charts of type of facility (*n* = 64), professional experience (*n* = 64) and case volume per year and institution (*n* = 64). Values given as percentage of respondents answering the respective question. Owing to rounding to whole numbers, percentages may not sum to exactly 100%. Absolute numbers are given in Supplementary Tables 1 and 10.

For defining older HNSCC patients, most respondents (47.0%) choose 70 years as an age threshold, with 19.3% and 18.1% choosing 65 and 75 years, respectively. Notably, the age threshold was significantly lower among non-European respondents. Of all respondents, 15.7% applied no specific age threshold with a higher proportion of European physicians using individual patient selection (25.0% vs 11.5%). For individual evaluation, performance status was widely assessed (92.9%), whereas Activities of Daily Living (ADL) and nutritional risk scores were used infrequently (13.1% and 2.4%). A comorbidity score was utilized by 32.1% and geriatric screening (mostly G8) by 25.0%.

For pretreatment imaging, contrast-enhanced CT (82.1%) and MRI (64.3%) of the head and neck formed the locoregional diagnostic backbone. While 40.5% of physicians reported staging with contrast-enhanced CT of the thorax and abdomen, PET/CT was routinely used by 61.9% in older patients (Fig. 2**, Supplementary Table 2**).

**Fig. 2:**
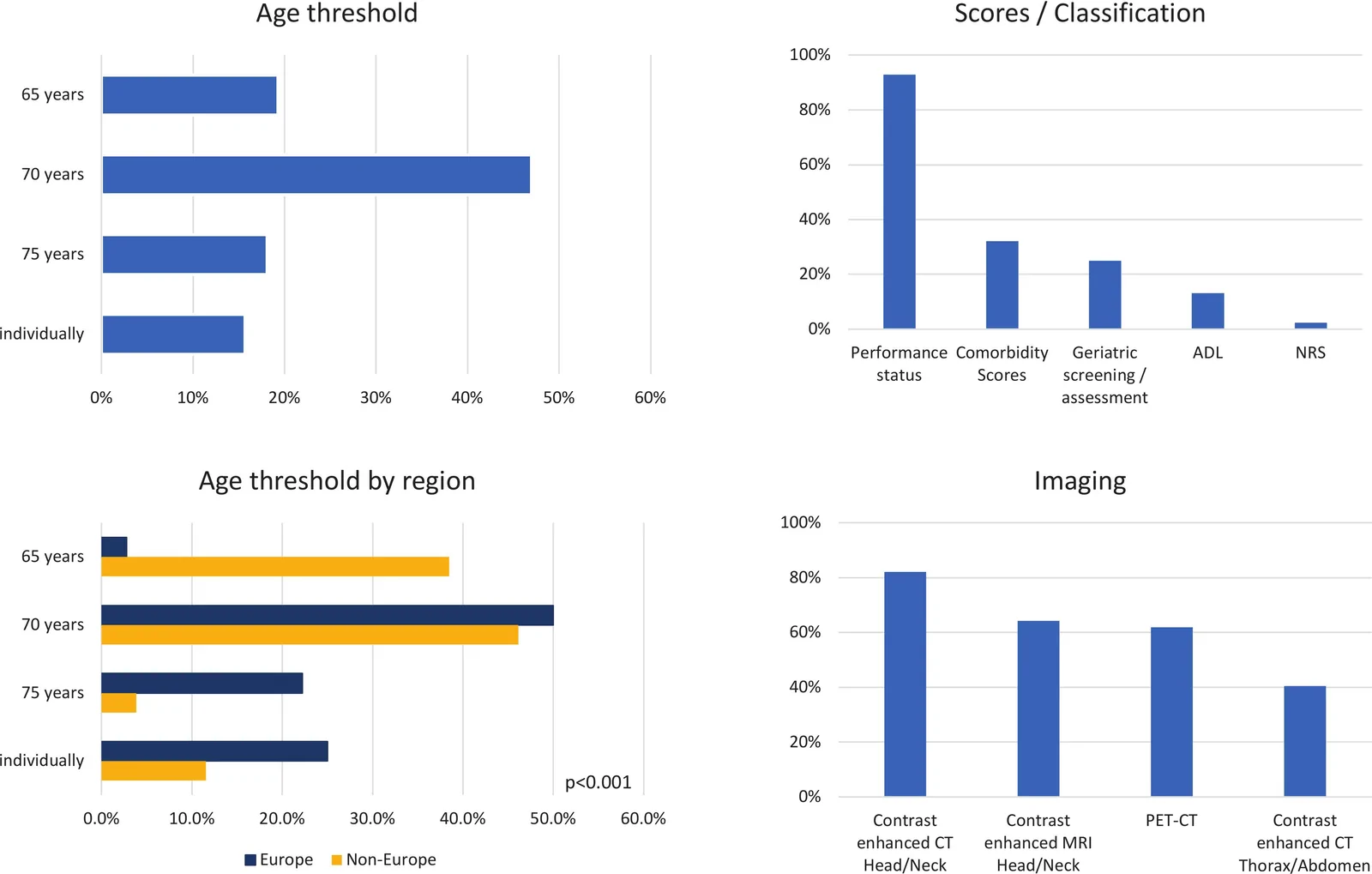
Bar charts of pretreatment work-up: age threshold to define an older HNSCC patient (*n* = 83), age threshold stratified by European versus non-European respondents (*n* = 36 and 26), routinely assessed scores or classifications (*n* = 84), and routine pretreatment imaging modalities (*n* = 84). Values given as percentage of respondents answering the respective question. Subgroup comparison by Fisher's exact test (*n* = 62, *p* < 0.001, Cramér's V = 0.51). (ADL, Activities of Daily Living; NRS, Nutritional Risk Screening).

HPV status was routinely assessed in older patients with oropharyngeal cancer (OPC) in 73.8% of institutions. P16 immunostaining, the common surrogate for HPV-related HNSCC, was routinely assessed in 54.8% of institutions, whereas 19.0% assessed both p16 and HPV DNA. 23.8% obtained HPV status upon request only, and 2.4% of institutions never assessed it. HPV testing differed significantly between European and non-European respondents (*p* = 0.004), with 42.3% of non-European physicians ordering no routine HPV status information in older patients with OPC. This mirrored the limited therapeutic relevance of HPV status. 80.3% reported no routine de-escalation for older patients with p16-positive OPC, and only 16.9% de-escalated within a clinical trial. Considering concomitant systemic therapy, 25.4% of respondents generally omitted chemotherapy in older patients with p16-positive OPC and an additional 17.9% did so in the adjuvant setting (Fig. 3**, Supplementary Table 3**).

**Fig. 3:**
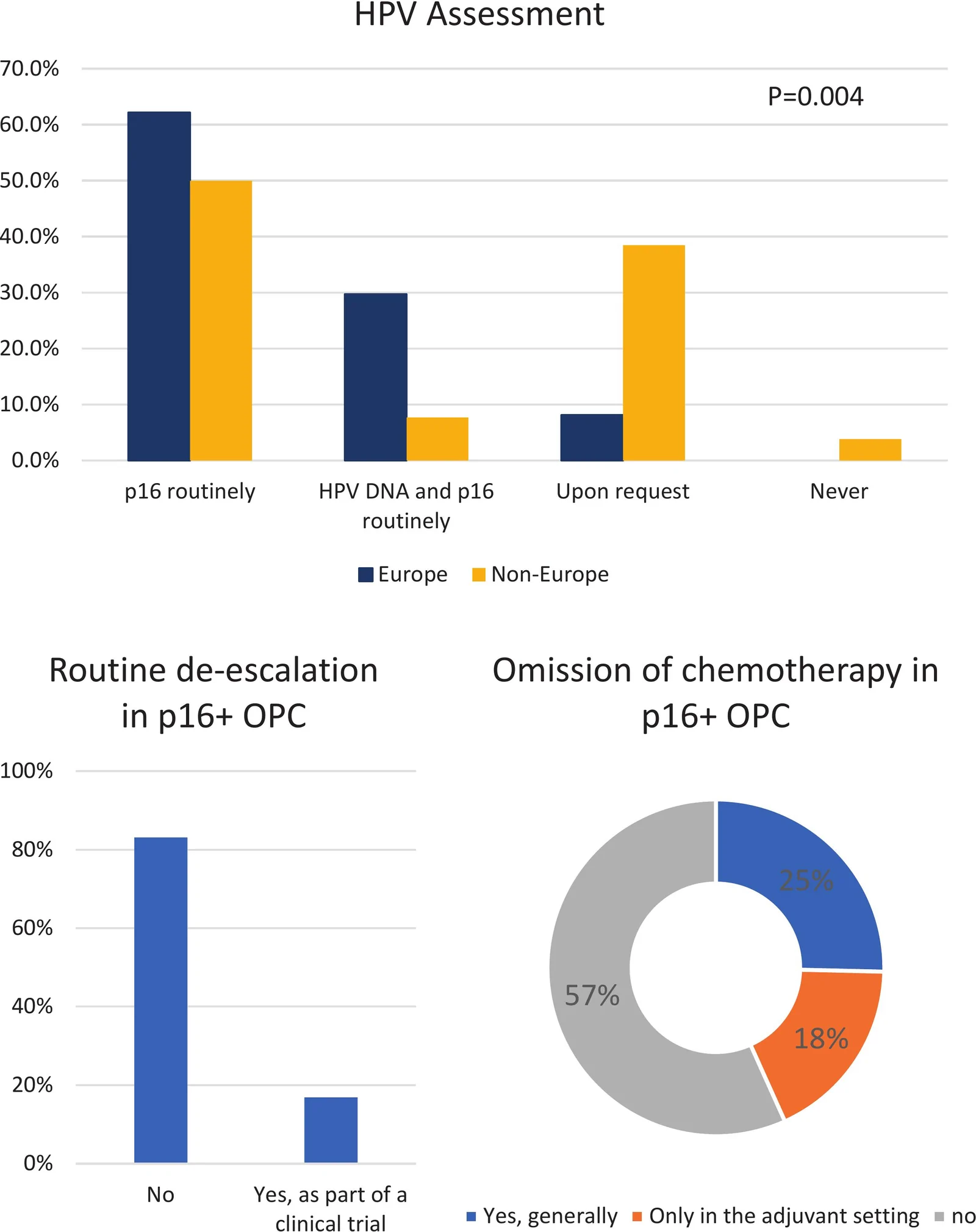
Bar charts and donut chart on HPV-related practice: HPV assessment in OPC stratified by European versus non-European respondents (*n* = 37 and 26), routine de-escalation strategies in p16-positive OPC (*n* = 71), and routine chemotherapy omission in p16-positive OPC (*n* = 67). Values given as percentage of respondents answering the respective question. Subgroup comparison by Fisher's exact test (*n* = 63, *p* = 0.004, Cramér's V = 0.44). (HPV, human papillomavirus; OPC, oropharyngeal cancer).

A relevant proportion of older HNSCC patients was treated in deviation from established guidelines. Most respondents (65.7%) estimated this proportion below 10% in their institution, but 31.4% assumed it up to 50%. In detail, 35.2% adapted target volumes for older patients with respect to age and comorbidities (18.3%) or T- and N-category (14.1%). HPV status rarely (2.8%) influenced target volume definition. The elective volume in the definitive setting mainly followed contouring guidelines (85.9%); only a small minority electively treated just the clinically involved neck side (5.6%) or the involved lymph node levels (1.4%). In the adjuvant setting, elective nodal delineation strategies appeared more heterogeneous. 65.7% of respondents suggested treating the bilateral neck, while 20.0% treated only the involved side of the neck or the involved lymph node levels (11.4%). Only 2.9% generally omitted the elective lymphatics in the adjuvant setting (Fig. 4**, Supplementary Table 4**).

**Fig. 4:**
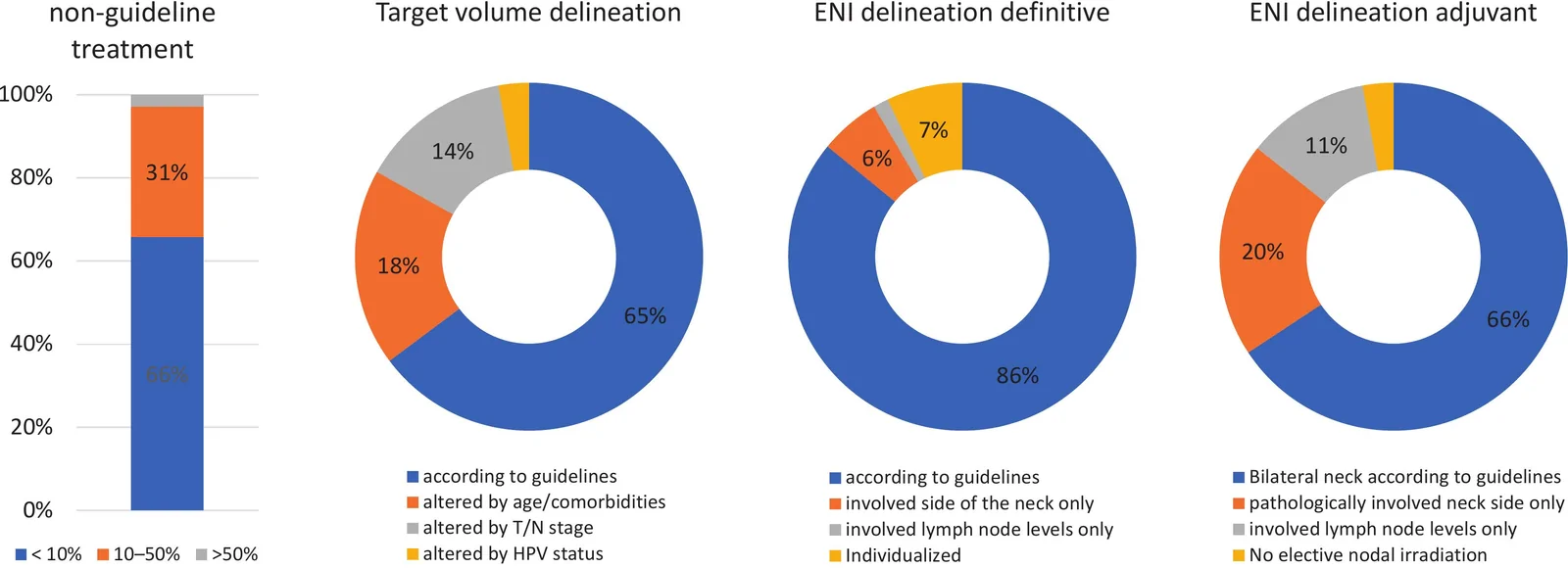
Stacked bar and donut charts on general therapy principles: proportion of patients treated outside current guidelines (*n* = 70), target volume delineation strategy (*n* = 71), and elective nodal irradiation in the definitive (*n* = 71) and adjuvant setting (*n* = 70). Values given as percentage of respondents answering the respective question. (ENI, elective nodal irradiation; HPV, human papillomavirus).

The benefit of concomitant chemotherapy in older HNSCC patients remains controversial. About half of respondents stated concomitant chemotherapy application also in older HNSCC patients if indicated and medically feasible, in both the definitive (52.2%) and adjuvant (50.7%) setting. The remaining suggested routinely omitting chemotherapy beyond a certain age ranging from 65 to 80 years (median 70 years). Factors considered in chemotherapy evaluation included performance status (97.0%), laboratory parameters such as blood count and renal function (86.6%), patient age (80.6%), comorbidity indices (68.7%), cardiac function (58.2%) and BMI/weight loss (50.7%). Geriatric screening was not among the predefined response options but was named under “other” by 6% of respondents. Weekly cisplatin was the most frequent regimen in both the definitive (59.7%) and adjuvant setting (62.7%). 2–3 cycles of high-dose cisplatin (10.4% and 9.0%), carboplatin monotherapy (4.5% and 9.0%), carboplatin/paclitaxel (4.5% and 4.5%), cetuximab (4.5% and 3.0%), and carboplatin/5-fluorouracil (1.5% and 4.5%) were reported as established but rare alternatives. Concerning cisplatin, a glomerular filtration rate or creatinine clearance below 60 mL/min was considered an ineligibility criterion for the majority of respondents (56.9%). Lower cut-offs of 50 and 40 mL/min were cited by 27.7% and 12.3%. Hearing loss was considered a cisplatin contraindication by 77.9% of physicians, with grade 2 (threshold shift >25 dB on two contiguous frequencies or hearing aid) being the most common threshold (41.2%). 22.1% still recommended cisplatin in older patients with hearing impairment. In case of chemotherapy ineligibility, 52.1% of respondents suggested alternative dose/fractionation schemes, most commonly hypofractionation (>2.5 Gy, 40.8%), followed by a reduced total dose (18.3%) and split-course regimens (14.1%). Acceleration (5.6%) and hyperfractionation (4.2%) were rarely recommended (Fig. 5**, Supplementary Table 5**).

**Fig. 5:**
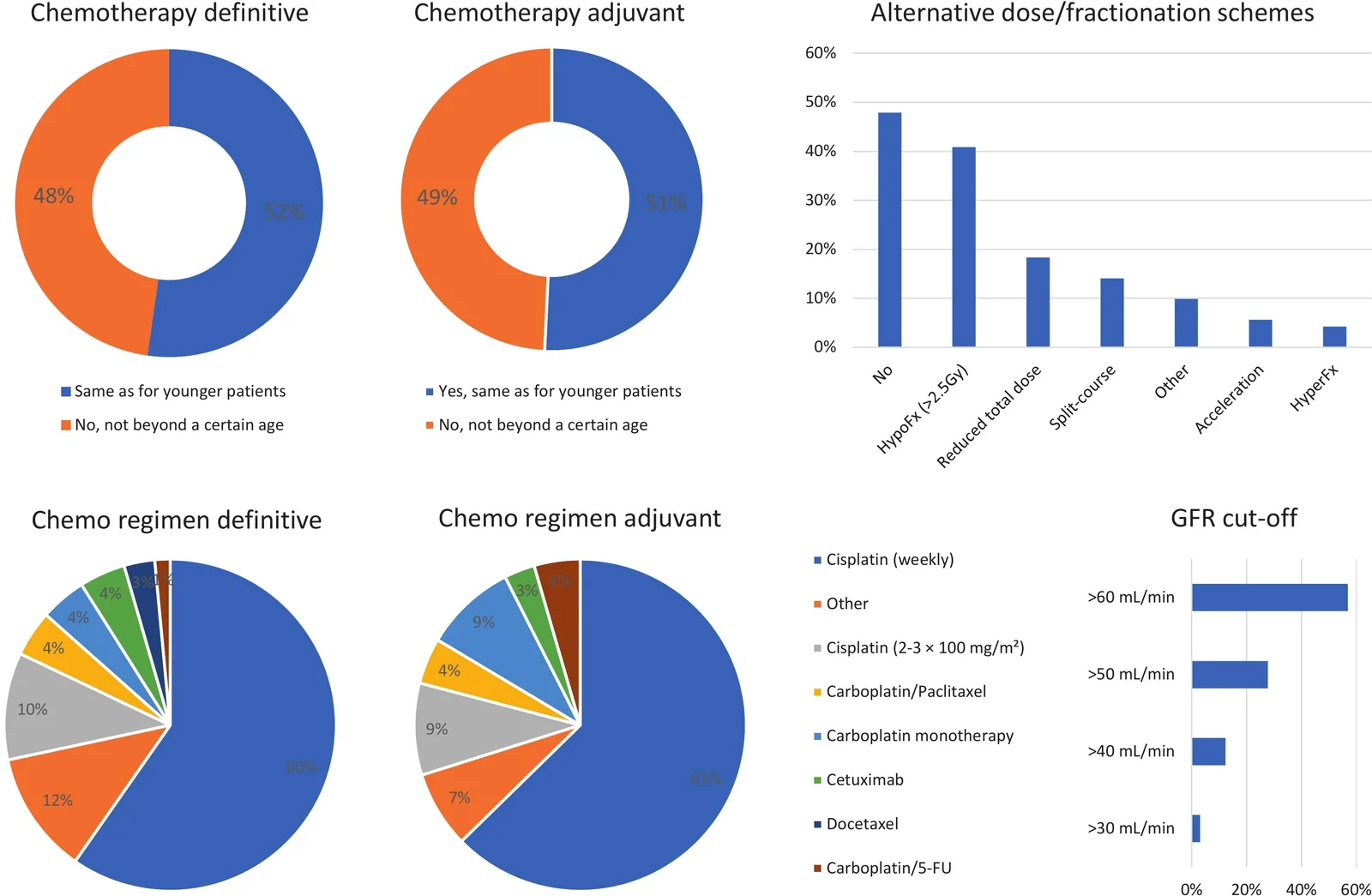
Donut charts and bar charts on chemotherapy practice: routine chemotherapy administration in the definitive and adjuvant setting (*n* = 67 each), distribution of chemotherapy regimens applied in the definitive and adjuvant setting (*n* = 67 each), alternative dose/fractionation schemes used in case of chemotherapy ineligibility (*n* = 71), and applied glomerular filtration rate cut-off for cisplatin eligibility (*n* = 65). Values given as percentage of respondents answering the respective question. (5-FU, 5-fluorouracil; GFR, glomerular filtration rate; HypoFx, hypofractionation; HyperFx, hyperfractionation).

A wide variety of supportive measures were offered to guide older HNSCC patients through curative (chemo)radiation. Nutritional counseling was almost ubiquitously provided (95.5%), while skin care consultations (59.1%), speech therapy (42.4%), and social work services (39.4%) were less often applied in clinical routine. A significant proportion of physicians (48.5%) routinely initiated prophylactic gastrostomy tube placement in older HNSCC patients, whereas central venous access was prescribed less frequently (25.8%). 30.3% routinely recommended physical therapy during treatment. Regarding the emerging role of prehabilitation in oncology, 21.9% of radiation oncologists routinely offered prehabilitation programs to older HNSCC patients undergoing curative radiotherapy, and another 17.2% after individual risk stratification. However, in the majority of institutions (60.9%), prehabilitation was not routinely offered for this patient cohort. Radiotherapy was typically suggested on an outpatient basis if feasible (60.6%), with 31.8% of physicians recommending inpatient admission for concomitant chemotherapy and only 7.6% recommending complete inpatient treatment for older HNSCC patients. During outpatient treatment, older patients were scheduled for weekly physician appointments (77.3%) or 2–3 times per week (13.6%). Follow-up mirrored that for younger patients in 69.7% of cases, and 22.7% of respondents stated to consult older patients more frequently (**Supplementary Table 6**).

Respondents were presented with two case studies. The first case described definitive treatment of a 76-year-old patient with locally advanced (T3) but clinically node-negative, well lateralized hypopharyngeal carcinoma who declined resection. For general treatment strategy, 85.7% recommended primary therapy with bilateral elective node irradiation, 12.7% recommended ipsilateral treatment only and 1.6% recommended treating only the primary tumor. 49.2% of physicians opted for concurrent chemoradiation and 50.8% for radiotherapy alone. Recommended dose to the primary tumor was 70 Gy (EQD2) for 87.3% of respondents and 66 Gy (EQD2) for 12.7%. 76.2% suggested conventional fractionation, whereas 12.7% recommended hypofractionation and 9.5% acceleration (Fig. 6**, Supplementary Table 7**).

**Fig. 6:**
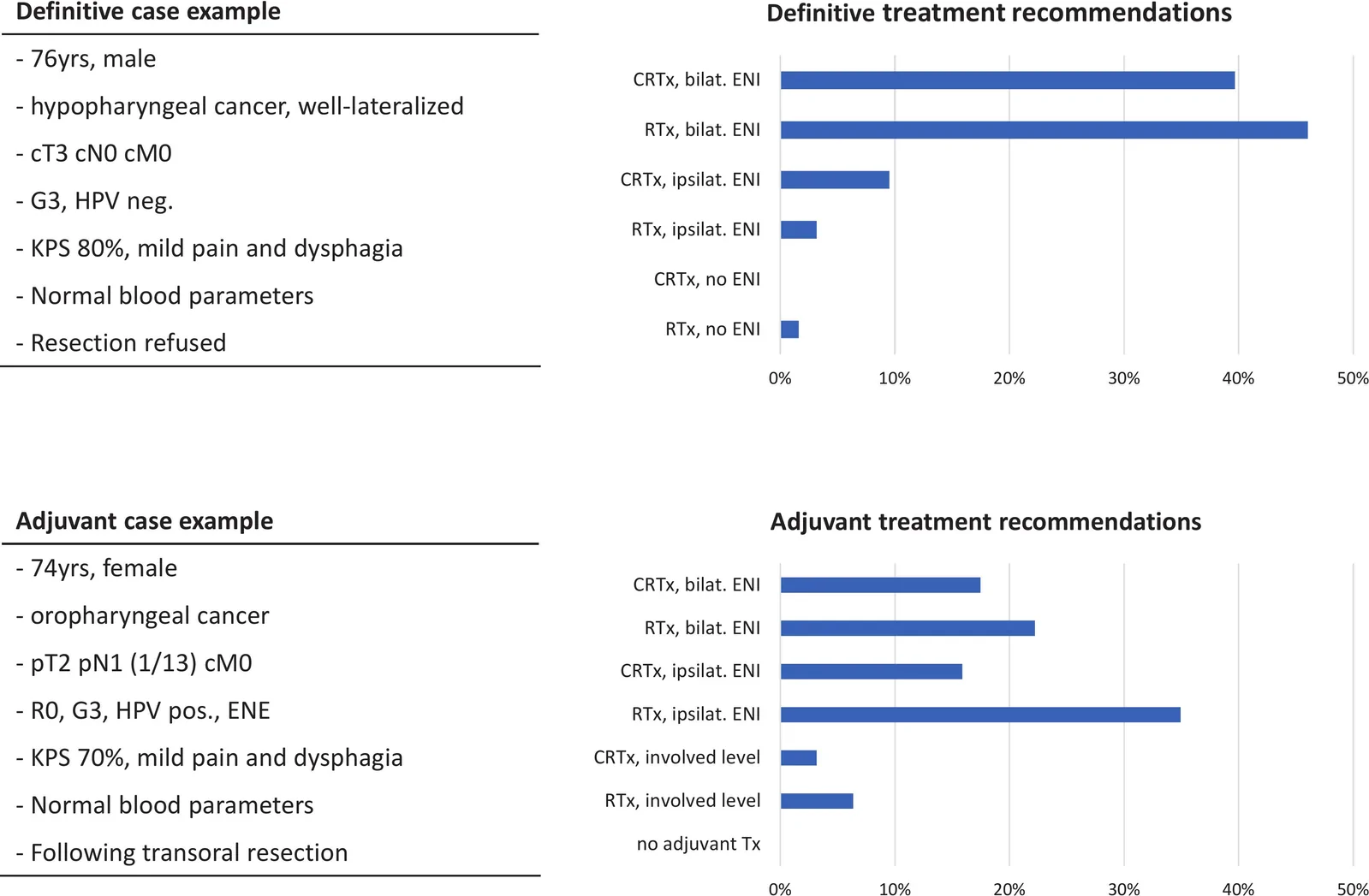
Representative cases of a typical older HNSCC patient in the definitive and in the adjuvant setting with key clinical features and recommended treatment approaches given as percentage of respondents answering the respective question (*n* = 63 for both cases). (CRTx, chemoradiation; ENE, extranodal extension; ENI, elective nodal irradiation; HPV, human papillomavirus; KPS, Karnofsky performance status; RTx, radiotherapy).

The second case presented a 74-year-old patient after transoral resection (R0) of a p16-positive OPC of the tonsil with extranodal extension of a single nodal metastasis. All respondents recommended adjuvant therapy. Most physicians (63.5%) suggested omitting concomitant chemotherapy, with only 36.5% opting for chemoradiation. Considering target volume definition, bilateral cervical irradiation was recommended by 39.7% of respondents, whereas a majority reduced volumes to either ipsilateral cervical irradiation (50.8%) or the involved nodal level only (9.5%). In line with guideline recommendations, total dose (EQD2) to the primary tumor region was determined at 60 Gy (63.5%) or 63–66 Gy (33.3%). For the region of extranodal extension, most recommended 63–66 Gy (73.0%), while some respondents suggested decreased doses of 50 Gy (7.9%). Conventional fractionation predominated (90.5%), with 7.9% of respondents favoring hypofractionation (Fig. 6**, Supplementary Table 8**).

## 4 Discussion

We conducted an international pattern-of-care survey on the radiation oncology management of older HNSCC patients. In summary, in our cohort of surveyed radiation oncologists several aspects appeared remarkably consistent, i.e. high adherence to guideline-based target volumes in the definitive setting, the frequent use of weekly cisplatin (established in the postoperative but not yet in the definitive setting), and caution regarding HPV-driven de-escalation. By contrast, considerable heterogeneity emerged in the implementation of geriatric concepts, the indication for concomitant systemic therapy, alternative fractionation, as well as target volume definition and de-escalation in the adjuvant setting.

The age threshold defining an older HNSCC patient was markedly heterogeneous in our survey, ranging from 65 to 75 years, with a significant proportion not applying any threshold at all. This mirrors the lack of a uniform definition and the differing cut-offs in trials and guidelines [9], [14]. The age threshold was significantly lower among non-European respondents, indicating that the chronological definition of an older patient is context-specific. Regional differences in life expectancy, healthcare structures and tumor epidemiology may contribute, yet none of these factors were captured in the survey, and this explanation remains hypothetical. Although performance status was nearly universally assessed (93%), structured comorbidity scoring (32%) and geriatric screening (25%, predominantly the G8) remained underused. This is in line with previous surveys from Italy and the EORTC [24], [25], yet considerably higher than the most recent comparable survey of German-speaking countries, where only 3% applied geriatric screening [23], suggesting gradual implementation of geriatric concepts in routine practice. The persistently low implementation also reflects conflicting evidence: While the G8 predicts overall survival and informs treatment decisions in older HNSCC patients undergoing curative (chemo-)radiotherapy [27], [28], the largest randomized trial of geriatric assessment-driven interventions in HNSCC, EGeSOR, showed no outcome benefit over standard of care [29]. Yet chronological age appeared to serve as a proxy for a fitness assessment that was infrequently performed in a structured manner by our respondents: 84% applied an age threshold to define the older patient and 81% considered age when deciding on chemotherapy, whereas the instruments capturing frailty and comorbidity were used significantly less often. For clinical decisions, age matters through the comorbidity, declining organ function and shifting preferences that accompany it, not as a threshold in itself [13], [15], [19], [20]. Geriatric oncology principles therefore call for individualized assessment of these domains rather than of chronological age [14], [15], which contrasts with the practice reported here. PET/CT was used more frequently than in earlier surveys [23], [24], [25], likely reflecting improved access, recognition for detecting second primaries/metastases [30] and incorporation into guidelines [31], [32].

HPV status for OPC was routinely assessed in 74% of institutions, with a marked geographical gradient. This may relate to regional differences in HPV-associated OPC incidence [33], cost considerations, and the limited therapeutic consequence of HPV. Although the prognostic value of HPV is well established also in older patients [34], [35], no de-escalation strategy is currently endorsed outside clinical trials regardless of age.

Adherence to guidelines for elective nodal volumes was high in the definitive setting (86%) exceeding the 73% of an earlier regional survey [23], and possibly reflecting the higher proportion of academic expert respondents in our cohort. In the adjuvant setting, by contrast, only 66% of respondents reported bilateral elective nodal irradiation, while 31% applied volume reduction; possibly reflecting the lower absolute recurrence risk and cumulative multimodal morbidity. This is backed by emerging evidence supporting unilateral neck irradiation in carefully selected patients [36], [37]. Comparably, the AIRO survey reported that 56% of Italian radiation oncologists routinely apply target volume reduction in older HNSCC patients [25].

Concomitant systemic therapy in older HNSCC patients remained an area of substantial heterogeneity, with around half of respondents routinely omitting chemotherapy beyond a self-defined age threshold of 65–80 years. This contrasts with the results of a large international registry, where an overall survival benefit for concomitant chemotherapy was observed in patients up to 80 years, especially in the absence of relevant comorbidities [13]. The marked variability in chemotherapy use is consistent with previous surveys: in the EORTC-led survey, chemoradiation use in older patients ranged from <5% to >40% across centers [24]. The Italian AIRO survey documented platinum-based chemoradiation in just 21% of curatively treated older patients [25]. The recent Japanese JCOG survey identified renal function and performance status as the dominant criteria rather than chronological age alone [26]. In this survey, weekly cisplatin was the most frequently reported regimen for both definitive (60%) and adjuvant treatments (63%), reflecting a clear shift from 3-weekly schedules and in line with the results from the Japanese survey, which advised against 3-weekly cisplatin in older or frail patients [26]. JCOG1008 established weekly cisplatin as non-inferior to 3-weekly cisplatin for adjuvant chemoradiation, with substantially lower rates of nephrotoxicity, ototoxicity and myelosuppression [38]; a profile particularly relevant for older patients. For the definitive setting results of the ConCERT and NRG-HN009 trials are still awaiting full publication [39]. The consistent use of weekly cisplatin is thus evidence-based in the postoperative setting, whereas in the definitive setting it reflects observed practice rather than established evidence. As renal function commonly declines with age, the applied GFR cut-off is of central importance for cisplatin eligibility in this population: 57% of respondents required a GFR above 60 mL/min, which excludes a relevant fraction of older patients. This threshold corresponds to the consensus definition of cisplatin unfitness developed for other entities [40], not to a formal guideline recommendation for HNSCC. However, dose-adjusted cisplatin administration down to 50 mL/min has been described [41], [42], again in other malignancies rather than in curative HNSCC chemoradiotherapy. No HNSCC-specific threshold has been established. The Japanese survey reported even more permissive cut-offs for weekly cisplatin (GFR <40 and < 50 mL/min for ≥65 and ≥ 70 years). As the methodology of renal function assessment varies across institutions, the questionnaire referred to glomerular filtration rate or creatinine clearance without specifying the method of determination. It should be acknowledged that estimates obtained by different formulas or measurements may differ substantially in the individual patient. Pre-existing hearing loss was considered a contraindication by 78% of respondents. Although no strict cut-offs have been formally endorsed by guidelines, this restraint reflects the importance of hearing preservation for quality of life, particularly social interactions. Since cisplatin-induced ototoxicity is substantially less pronounced with weekly schedules [43], audiometric monitoring may preserve cisplatin eligibility in this population.

Hypofractionation, used in chemotherapy ineligibility, has received limited attention in earlier surveys [23], [24], [25], yet is substantially informed by two recently published RCTs. ELAN-RT represents a treatment de-intensification through a hypofractionated split-course regimen for frail older patients, achieving good locoregional remission and lower acute toxicity, albeit inferior OS [22]. By contrast, the HYPNO trial demonstrated non-inferiority of a 20-fraction definitive regimen for 3-year locoregional control and late toxicity in younger patients [44], which could become an option for fitter older patients, reducing treatment times. Although hypofractionation and a reduced total dose were the most frequently reported alternatives in our survey, the specific schedules were not captured. Given the range of possible regimens and combinations, from moderately hypofractionated definitive treatment through definitive split-course to palliative hypofractionated regimens, no single hypofractionated schedule can be considered established within our cohort. The results do, however, underline the clinical heterogeneity and the absence of an evidence-based de-escalation standard for this population.

Supportive care patterns were heterogeneous and may hold underused potential. Prophylactic gastrostomy tube placement, an ongoing debate, was suggested routinely by 49% of respondents. Randomized trials show reduced weight loss and improved short-term quality of life, but no clear oncological benefit and a potential for long-term tube dependence [45], [46]. Sarcopenia and reduced physiological reserve, both more prevalent in older patients, may strengthen the rationale for prophylactic placement, whereas age itself does not. Yet prolonged enteral feeding also risks accelerated swallowing dysfunction. Notably, 61% of respondents reported that prehabilitation was not routinely offered, despite emerging evidence supporting structured prehabilitation programs in HNSCC and other malignancies [47], [48]. Speech and swallowing therapy were offered routinely by only 42%, contrasting with the value older patients place on functional preservation [20] and with guideline recommendations for swallowing assessment and therapy throughout HNSCC treatment [49], [50]. The questionnaire asked for measures routinely provided and did not distinguish between deliberate non-use and lack of availability due to different health care systems and institutional resources. The reported proportions therefore reflect delivered practice rather than physician preference.

The two example cases allow translating the aggregate response patterns into concrete clinical decisions. The definitive case (76 years, hypopharyngeal cT3 cN0, KPS 80) showed high guideline adherence with a markedly homogeneous (86%) use of bilateral elective nodal irradiation, normofractionation and standard prescription doses. Considering the substantial risk of occult bilateral nodal involvement in hypopharyngeal carcinoma, volume de-escalation in this case would carry a clearly increased risk of recurrence. The near-equal split between primary chemoradiation (49%) and radiotherapy alone (51%) reflects, on one hand, the evidence base from the SENIOR cohort supporting concomitant chemotherapy in a 76-year-old patient with good performance status [13], and on the other hand the broader restraint regarding chemotherapy in older patients observed throughout our survey.

The adjuvant case (74 years, p16-positive OPC, pT2 pN1 with ENE, KPS 70) showed substantially greater heterogeneity. 63% of respondents would omit concomitant chemotherapy despite ENE, the strongest indication for postoperative chemoradiation [6], [7], and 60% reduced elective volumes to the ipsilateral neck (51%) or to the involved nodal level only (10%). A comparable adjuvant case in the trinational survey of German-speaking countries revealed similar heterogeneity [23]. The two deviations are, however, of a different nature. Volume reduction represents individualization within an evolving evidence base in selected patients [36], [37], whereas omission of chemotherapy in the presence of ENE represents de-intensification driven by age and anticipated tolerability without prospective validation. With a borderline performance status, the trade-off between expected oncological benefit and cumulative multimodal toxicity becomes genuinely difficult, and a majority of respondents resolved it in favor of toxicity avoidance.

Our study faces certain limitations. Recruitment through the ESTRO mailing list and social media may have favored academic and interested participants, limiting generalizability. As the survey link was open and non-personalized, a conventional response rate could not be calculated and repeated participation could not be technically excluded. The absolute response number, while comparable to previous surveys conducted within ESTRO, impedes subgroup analyses. In particular, the non-European group was heterogeneous, dominated by respondents practicing in Asia (**Supplementary Table 10**). Surveys inherently capture stated rather than observed practice and are subject to recall and social desirability biases. National and regional discrepancies in public health systems, reimbursement, or availability of radiotherapy facilities can also play a crucial role in decisions but could not be captured here. Finally, the survey was designed before the approval of immunotherapy for the curative setting and the publication of the randomized proton therapy trials [51], [52], [53], [54]. Implementation of these developments in treatment of older HNSCC patients was therefore not addressed. Despite these limitations, this international survey on behalf of the ESTRO Head and Neck Focus Group provides valuable insight into the current radiotherapeutic management of older HNSCC patients in light of the recently emerging evidence base. With 41% of respondents practicing outside Europe and a high proportion of board-certified academic experts, our cohort of surveyed radiation oncologists offers broader geographic scope and clinical expertise than previous regional surveys. We identified areas of relative consensus and adherence to evidence-based principles as well as clinically relevant heterogeneity that warrants future evidence-generating efforts and consensus development for this growing patient group.

## CRediT authorship contribution statement

**Erik Haehl:** Writing – original draft, Methodology, Investigation, Formal analysis, Data curation, Conceptualization. **Alexander Rühle:** Writing – review & editing, Methodology, Conceptualization. **Panagiotis Balermpas:** Writing – review & editing, Supervision. **Jon Cacicedo:** Writing – review & editing, Supervision, Conceptualization. **Franziska Walter:** Writing – review & editing. **Claus Belka:** Writing – review & editing. **Nils Henrik Nicolay:** Writing – review & editing, Supervision, Methodology, Conceptualization.

## Ethics declaration

**Informed consent and patient details**: The authors have NOT obtained informed consent from participants or their legal representatives. Written informed consent was not obtained, as the survey was anonymous and addressed physicians only. Respondents voluntarily opened the invitation link distributed via email or social media and actively submitted the questionnaire at the end of the online survey. Completion of the questionnaire was therefore considered to imply consent to participate.

**Studies in Human**: This study was performed in compliance with relevant laws, regulatory frameworks and guidelines where the research took place. Ethics committee approval was not required under relevant laws and institutional guidelines. This was a patterns-of-care survey among radiation oncologists. Participation was voluntary and anonymous. No personal or identifying data of the participants were collected, and no patient-related data were requested in the survey. Therefore, ethics committee approval was not required.

## Declaration of generative AI and AI-assisted technologies in the writing process

During the preparation of this work, the author(s) used Claude (Anthropic) in order to improve the language and readability of the manuscript. After using this tool/service, the author(s) reviewed and edited the content as needed and take(s) full responsibility for the content of the published article.

## Declaration of competing interest

The authors declare that they have no known competing financial interests or personal relationships that could have appeared to influence the work reported in this paper.
