# Supplementary material for: Clinical practice patterns for radiotherapy in older patients with head and neck cancer: Results of the ESTRO head and neck focus group patterns-of-care survey
Source: Clin Transl Radiat Oncol. 2026 Nov 4;62:101351. doi: 10.1016/j.ctro.2026.101351 (PMC13769551; doi:10.1016/j.ctro.2026.101351)
Supplement: Supplementary file 1 [file mmc1.docx]

**Supplementary Table 1.** Respondent and institutional characteristics (Figure 1)

| **Category** | **% (n)** |
| --- | --- |
| **Geographic region — by continent (Q34; n = 63)** | |
| Europe | 58.7% (37) |
| Asia | 31.7% (20) |
| North America | 6.3% (4) |
| Africa | 1.6% (1) |
| Australia | 1.6% (1) |
| **European respondents — by sub-region (Q34; % of European respondents)** | |
| Northern Europe | 18.9% (7) |
| Western Europe | 45.9% (17) |
| Southern Europe | 16.2% (6) |
| Eastern Europe | 18.9% (7) |
| **Type of institution (Q31; n = 64)** | |
| Academic hospital | 76.6% (49) |
| General hospital | 20.3% (13) |
| Outpatient facility | 3.1% (2) |
| **Professional role (Q32; n = 64)** | |
| Resident | 3.1% (2) |
| Board-certified radiation oncologist | 51.6% (33) |
| Board-certified with more than 10 years of experience | 45.3% (29) |
| **Annual elderly HNSCC caseload at institution (Q33; n = 64)** | |
| < 10 | 3.1% (2) |
| 10–50 | 46.9% (30) |
| 51–100 | 12.5% (8) |
| > 100 | 37.5% (24) |

**Supplementary Table 2.** Definition of “older”, geriatric assessment, and staging work-up (Figure 2)

| **Category** | **% (n)** |
| --- | --- |
| **Age threshold used to classify a patient as elderly (Q1; n = 83)** | |
| 65 years | 19.3% (16) |
| 70 years | 47.0% (39) |
| 75 years | 18.1% (15) |
| No specific value / individually | 15.7% (13) |
| **Scores or classifications routinely assessed — multiple answers possible (Q2; n = 84)** | |
| Performance status (e.g., ECOG/WHO, Karnofsky Index) | 92.9% (78) |
| Comorbidity scores (e.g., Charlson Comorbidity Index, ACE-27) | 32.1% (27) |
| Geriatric screening / assessment | 25.0% (21) |
| ADL | 13.1% (11) |
| NRS | 2.4% (2) |
| **Imaging modalities routinely used for staging — multiple answers possible (Q3; n = 84)** | |
| Contrast-enhanced CT head/neck | 82.1% (69) |
| Contrast-enhanced MRI head/neck | 64.3% (54) |
| PET-CT | 61.9% (52) |
| Contrast-enhanced CT thorax/abdomen | 40.5% (34) |

**Supplementary Table 2 (continued).** Age threshold to define elderly patients, by world region (Q1)

| **Category** | **Europe, % (n)** | **Non-Europe, % (n)** |
| --- | --- | --- |
| 65 years | 2.8% (1) | 38.5% (10) |
| 70 years | 50.0% (18) | 46.2% (12) |
| 75 years | 22.2% (8) | 3.8% (1) |
| Individually | 25.0% (9) | 11.5% (3) |
| *Europe n=36; Non-Europe n=26; total n=62; χ² p=0.001; Fisher's exact p=0.0009; Cramér's V=0.51* | | |

**Supplementary Table 3.** HPV testing and HPV-driven treatment decisions (Figure 3)

| **Category** | **% (n)** |
| --- | --- |
| **HPV status assessment in oropharyngeal cancers (Q4; n = 84)** | |
| p16 routinely | 54.8% (46) |
| HPV DNA and p16 routinely | 19.0% (16) |
| Upon request | 23.8% (20) |
| Never | 2.4% (2) |
| **Routine de-escalation of therapy in p16-positive oropharyngeal cancer (Q5; n = 71)** | |
| No | 80.3% (57) |
| Yes, as part of a clinical trial | 16.9% (12) |
| Yes, routinely | 2.8% (2) |
| **Routine omission of chemotherapy in p16-positive oropharyngeal cancer (Q13; n = 67)** | |
| Yes, generally | 25.4% (17) |
| Only in the adjuvant setting | 17.9% (12) |
| No | 56.7% (38) |

**Supplementary Table 3 (continued).** HPV status assessment, by world region (Q4)

| **Category** | **Europe, % (n)** | **Non-Europe, % (n)** |
| --- | --- | --- |
| p16 routinely | 62.2% (23) | 50.0% (13) |
| HPV DNA and p16 routinely | 29.7% (11) | 7.7% (2) |
| Upon request | 8.1% (3) | 38.5% (10) |
| Never | 0.0% (0) | 3.8% (1) |
| *Europe n=37; Non-Europe n=26; total n=63; χ² p=0.007; Fisher's exact p=0.004; Cramér's V=0.44* | | |

**Supplementary Table 4.** Therapy principles: target volume and elective nodal irradiation (Figure 4)

| **Category** | **% (n)** |
| --- | --- |
| **Proportion of elderly patients treated in deviation from standard guidelines (Q10; n = 70)** | |
| < 10% | 65.7% (46) |
| 10–50% | 31.4% (22) |
| > 50% | 2.9% (2) |
| **Target volume delineation (Q6; n = 71)** | |
| According to guidelines | 64.8% (46) |
| Altered by age / comorbidities | 18.3% (13) |
| Altered by T/N stage | 14.1% (10) |
| Altered by HPV status | 2.8% (2) |
| **Elective nodal irradiation — definitive setting (Q7; n = 71)** | |
| According to to guidelines | 85.9% (61) |
| Involved side of the neck only | 5.6% (4) |
| Involved lymph node levels only | 1.4% (1) |
| Individualized based on pre-treatment imaging | 7.0% (5) |
| **Elective nodal irradiation — adjuvant setting (Q8; n = 70)** | |
| Bilateral neck, according to guidelines | 65.7% (46) |
| Pathologically involved neck side only | 20.0% (14) |
| Involved lymph node levels only | 11.4% (8) |
| No elective nodal irradiation | 2.9% (2) |

**Supplementary Table 5.** Systemic therapy (Figure 5)

| **Category** | **% (n)** |
| --- | --- |
| **Chemotherapy in the definitive setting (Q11; n = 67)** | |
| Yes, same as for younger patients (if medically feasible) | 52.2% (35) |
| No, not beyond a certain age | 47.8% (32) |
| **Chemotherapy in the adjuvant setting (if indicated) (Q12; n = 67)** | |
| Yes, same as for younger patients (if medically feasible) | 50.7% (34) |
| No, not beyond a certain age | 49.3% (33) |
| **Factors considered when deciding on chemotherapy — multiple answers possible (Q14; n = 67)** | |
| Age | 80.6% (54) |
| Performance status | 97.0% (65) |
| Comorbidity index | 68.7% (46) |
| Laboratory parameters (e.g., renal function, hepatic function, CBC) | 86.6% (58) |
| Cardiac function (ECG) | 58.2% (39) |
| BMI / weight loss | 50.7% (34) |
| Other | 11.9% (8) |
| *Free-text answers under "other" included geriatric screening (n=4)* | |
| **Most commonly used regimen — definitive setting (Q15; n = 67)** | |
| Cisplatin (30–40 mg/m² weekly) | 59.7% (40) |
| Cisplatin (2–3 × 100 mg/m²) | 10.4% (7) |
| Carboplatin monotherapy | 4.5% (3) |
| Carboplatin/Paclitaxel | 4.5% (3) |
| Cetuximab | 4.5% (3) |
| Docetaxel | 3.0% (2) |
| Carboplatin/5-FU | 1.5% (1) |
| Mitomycin C/5-FU | 0.0% (0) |
| Mitomycin monotherapy | 0.0% (0) |
| Other | 11.9% (8) |
| **Most commonly used regimen — adjuvant setting (Q16; n = 67)** | |
| Cisplatin (30–40 mg/m² weekly) | 62.7% (42) |
| Cisplatin (2–3 × 100 mg/m²) | 9.0% (6) |
| Carboplatin monotherapy | 9.0% (6) |
| Carboplatin/5-FU | 4.5% (3) |
| Carboplatin/Paclitaxel | 4.5% (3) |
| Cetuximab | 3.0% (2) |
| Docetaxel | 0.0% (0) |
| Mitomycin C/5-FU | 0.0% (0) |
| Mitomycin monotherapy | 0.0% (0) |
| Other | 7.5% (5) |
| **GFR / creatinine clearance cut-off for cisplatin eligibility (Q17; n = 65)** | |
| > 60 mL/min | 56.9% (37) |
| > 50 mL/min | 27.7% (18) |
| > 40 mL/min | 12.3% (8) |
| > 30 mL/min | 3.1% (2) |
| **Hearing loss considered a cisplatin contraindication (Q18; n = 68)** | |
| Yes, ≥ grade 1 (threshold shift 15–25 dB, two contiguous frequencies) | 22.1% (15) |
| Yes, ≥ grade 2 (threshold shift > 25 dB, two contiguous frequencies or hearing aid) | 41.2% (28) |
| Yes, ≥ grade 3 (threshold shift > 25 dB, three contiguous frequencies) | 14.7% (10) |
| No | 22.1% (15) |
| **Alternative dose / fractionation schemes if chemotherapy is contraindicated — multiple answers possible (Q9; n = 71)** | |
| No | 47.9% (34) |
| Hypofractionation (> 2.5 Gy) | 40.8% (29) |
| Reduced total dose | 18.3% (13) |
| Split-course | 14.1% (10) |
| Other | 9.9% (7) |
| Acceleration (> 5 fractions per week) | 5.6% (4) |
| Hyperfractionation | 4.2% (3) |

**Supplementary Table 6.** Supportive care and follow-up

| **Category** | **% (n)** |
| --- | --- |
| **Supportive measures routinely provided during curative treatment — multiple answers possible (Q19; n = 66)** | |
| Nutritional counseling | 95.5% (63) |
| Skin care consultation | 59.1% (39) |
| Prophylactic gastrostomy tube placement (PEG) | 48.5% (32) |
| Speech therapy | 42.4% (28) |
| Social work services | 39.4% (26) |
| Physical therapy | 30.3% (20) |
| Central venous access | 25.8% (17) |
| Other | 12.1% (8) |
| **Routine prehabilitation prior to (chemo)radiotherapy (Q20; n = 64)** | |
| No | 60.9% (39) |
| For definitive therapy | 17.2% (11) |
| Individually | 17.2% (11) |
| For adjuvant therapy preoperatively | 4.7% (3) |
| **Management during radiation therapy (treatment setting) (Q21; n = 66)** | |
| Outpatient if medically feasible | 60.6% (40) |
| Outpatient with inpatient admission for chemotherapy | 31.8% (21) |
| Complete inpatient treatment | 7.6% (5) |
| **Physician visit interval during radiation therapy (Q22; n = 66)** | |
| Weekly | 77.3% (51) |
| 2–3 times per week | 13.6% (9) |
| As needed | 6.1% (4) |
| Daily | 3.0% (2) |
| **Follow-up schedule compared with younger patients (Q23; n = 66)** | |
| Same | 69.7% (46) |
| More frequent | 22.7% (15) |
| Less frequent | 7.6% (5) |

**Supplementary Table 7.** Clinical vignette 1 — definitive setting (Figure 6)

*Case: 76-year-old patient; well-lateralized hypopharyngeal cancer; cT3 cN0 cM0; G3, p16/HPV-negative; KPS 80% with mild pain and dysphagia; normal blood parameters; surgery declined.*

| **Category** | **% (n)** |
| --- | --- |
| **Recommended treatment strategy (Q24; n = 63)** | |
| Primary radiotherapy with bilateral cervical nodal irradiation | 46.0% (29) |
| Primary chemoradiotherapy with bilateral cervical nodal irradiation | 39.7% (25) |
| Primary chemoradiotherapy with ipsilateral nodal irradiation | 9.5% (6) |
| Primary radiotherapy with ipsilateral nodal irradiation | 3.2% (2) |
| Primary radiotherapy of the primary tumor only | 1.6% (1) |
| Primary chemoradiotherapy of the primary tumor only | 0.0% (0) |
| **Recommended total dose to the primary tumor region (Q25; n = 63)** | |
| 70 Gy (EQD2) | 87.3% (55) |
| 66 Gy (EQD2) | 12.7% (8) |
| 60 Gy (EQD2) | 0.0% (0) |
| < 60 Gy (EQD2) | 0.0% (0) |
| **Recommended fractionation concept (Q26; n = 63)** | |
| Conventional (normofractionated) | 76.2% (48) |
| Hypofractionated | 12.7% (8) |
| Accelerated | 9.5% (6) |
| Hyperfractionated | 1.6% (1) |

**Supplementary Table 8.** Clinical vignette 2 — adjuvant setting (Figure 6)

*Case: 74-year-old patient; oropharyngeal cancer; pT2 pN1 (1/13) cM0; R0, G3, p16/HPV-positive, with extranodal extension; KPS 70% with mild pain and dysphagia; normal blood parameters; after trans-oral resection.*

| **Category** | **% (n)** |
| --- | --- |
| **Recommended treatment strategy (Q27; n = 63)** | |
| Adjuvant radiotherapy with ipsilateral nodal irradiation | 34.9% (22) |
| Adjuvant radiotherapy with bilateral cervical nodal irradiation | 22.2% (14) |
| Adjuvant chemoradiotherapy with bilateral cervical nodal irradiation | 17.5% (11) |
| Adjuvant chemoradiotherapy with ipsilateral nodal irradiation | 15.9% (10) |
| Adjuvant radiotherapy of the primary site and involved nodal levels | 6.3% (4) |
| Adjuvant chemoradiotherapy of the primary site and involved nodal levels | 3.2% (2) |
| No adjuvant therapy, close imaging follow-up | 0.0% (0) |
| **Recommended total dose to the primary tumor region (Q28; n = 63)** | |
| 60 Gy (EQD2) | 63.5% (40) |
| 63–66 Gy (EQD2) | 33.3% (21) |
| 50 Gy (EQD2) | 3.2% (2) |
| < 50 Gy (EQD2) | 0.0% (0) |
| **Recommended dose to the region of extranodal extension (Q29; n = 63)** | |
| 63–66 Gy (EQD2) | 73.0% (46) |
| 60 Gy (EQD2) | 19.0% (12) |
| 50 Gy (EQD2) | 7.9% (5) |
| < 50 Gy (EQD2) | 0.0% (0) |
| **Recommended fractionation concept (Q30; n = 63)** | |
| Conventional (normofractionated) | 90.5% (57) |
| Hypofractionated | 7.9% (5) |
| Accelerated | 1.6% (1) |
| Hyperfractionated | 0.0% (0) |
