# Supplementary material for: Clinical practice patterns for radiotherapy in older patients with head and neck cancer: Results of the ESTRO head and neck focus group patterns-of-care survey
Source: Clin Transl Radiat Oncol. 2026 Nov 4;62:101351. doi: 10.1016/j.ctro.2026.101351 (PMC13769551; doi:10.1016/j.ctro.2026.101351)
Supplement: Supplementary file 2 [file mmc2.docx]

**Supplementary Table 9.** Full questionnaire and item-level response numbers

*The questionnaire comprised 34 items: 4 on pretreatment assessment and staging, 14 on treatment parameters, 5 on supportive care, 7 case-specific items based on two clinical example cases, and 4 on respondents’ and institutional characteristics. Items were single-choice or multiple-response; several offered an additional free-text field, as indicated. All 85 radiation oncologists entering the survey are included in the denominator of the response rate. Individual items could be skipped. Percentages reported in the manuscript and in Supplementary Tables 1–8 refer to the number of respondents answering the respective item.*

| **Item** | **Question (verbatim)** | **Response options (verbatim)** | **Type** | **Answered, n (%)** | **Skipped, n** |
| --- | --- | --- | --- | --- | --- |
| **Pretreatment assessment and staging (items 1–4)** | | | | | |
| 1 | What age threshold do you typically use to classify a patient with HNSCC as elderly? | 65 years  70 years  75 years  No specific value / individually | Single choice | 83 (97.6) | 2 |
| 2 | Which scores or classifications do you routinely assess in elderly head and neck cancer patients? (Multiple answers possible) | Performance status (e.g., ECOG/WHO, Karnofsky Index)  Comorbidity Scores (e.g., Charlson Comorbidity Index, ACE-27)  ADL  NRS  Geriatric screening / assessment (please specify) | Multiple response  *incl. free-text field* | 84 (98.8) | 1 |
| 3 | Which imaging modalities are routinely used for staging in elderly head and neck cancer patients? (Multiple answers possible) | Contrast enhanced CT Head/Neck  Contrast enhanced MRI Head/Neck  Contrast enhanced CT Thorax/Abdomen  PET-CT | Multiple response | 84 (98.8) | 1 |
| 4 | In oropharyngeal cancers, is HPV status routinely assessed in elderly patients at your institution? | p16 routinely  HPV DNA and p16 routinely  Upon request  Never | Single choice | 84 (98.8) | 1 |
| **Treatment parameters (items 5–18)** | | | | | |
| 5 | In p16-positive oropharyngeal tumors, is a de-escalation of therapy (e.g., reduced target volume or dose) implemented for elderly patients? | No  Yes, as part of a clinical trial  Yes, routinely (please specify) | Single choice  *incl. free-text field* | 71 (83.5) | 14 |
| 6 | Do you apply an adapted target volume definition for elderly patients? | No, routinely based on SOPs/contouring guidelines (e.g., Grégoire et al, 2018)  Yes, adapted based on age and comorbidities  Yes, adapted based on T/N stage  Yes, adapted based on HPV status | Single choice | 71 (83.5) | 14 |
| 7 | In the definitive setting, which elective lymph node target volumes do you routinely irradiate in elderly patients? | According to consensus guidelines (e.g., Biau et al, 2019)  Only the clinically involved side of the neck  Only the involved lymph node levels  Individualized based on pre-treatment imaging (please specify) | Single choice  *incl. free-text field* | 71 (83.5) | 14 |
| 8 | In the adjuvant setting, which elective lymph node target volumes do you routinely irradiate in elderly patients? | No elective nodal irradiation  Only the involved lymph node levels  Only the pathologically involved neck side  Bilateral cervical lymphatic drainage according to guidelines | Single choice | 70 (82.4) | 15 |
| 9 | Do you routinely apply alternative dose/fractionation schemes in elderly patients in case of chemotherapy contraindications? (Multiple answers possible) | No  Split-course  Hypofractionation (>2,5Gy)  Hyperfractionation  Acceleration (>5 fractions per week)  Reduced total dose  Other (please specify) | Multiple response  *incl. free-text field* | 71 (83.5) | 14 |
| 10 | What proportion of elderly head and neck cancer patients at your institution are treated in deviation from standard guidelines (e.g., NCCN, ESTRO consensus, S3)? | < 10%  10–50%  >50% | Single choice | 70 (82.4) | 15 |
| 11 | Do you routinely administer chemotherapy to elderly patients in the definitive setting? | Yes, same as for younger patients (if medically feasible)  No, not beyond a certain age (please specify the age) | Single choice  *incl. free-text field* | 67 (78.8) | 18 |
| 12 | Do you routinely administer chemotherapy to elderly patients in the adjuvant setting if indicated? | Yes, same as for younger patients (if medically feasible)  No, not beyond a certain age (please specify the age) | Single choice  *incl. free-text field* | 67 (78.8) | 18 |
| 13 | Do you routinely omit chemotherapy for p16-positive oropharyngeal cancers in elderly patients? | Yes, generally  Only in the adjuvant setting  no | Single choice | 67 (78.8) | 18 |
| 14 | Which of the following factors do you consider when deciding on chemotherapy in elderly patients? (Multiple answers possible) | Age  Performance Status  Comorbidity Index  Laboratory parameters (e.g., renal function, hepatic function, CBC)  Cardiac function (ECG)  BMI / weight loss  Other (please specify) | Multiple response  *incl. free-text field* | 67 (78.8) | 18 |
| 15 | Which chemotherapy regimen do you most commonly use in the definitive setting in elderly patients? | Cisplatin (2-3 × 100 mg/m²)  Cisplatin (30-40 mg/m² weekly)  Carboplatin/5-FU  Carboplatin/Paclitaxel  Carboplatin monotherapy  Mitomycin C/5-FU  Mitomycin monotherapy  Docetaxel  Cetuximab  Other (please specify) | Single choice  *incl. free-text field* | 67 (78.8) | 18 |
| 16 | Which chemotherapy regimen do you most commonly use in the adjuvant setting in elderly patients (if medically indicated)? | Cisplatin (2-3 × 100 mg/m²)  Cisplatin (30-40 mg/m² weekly)  Carboplatin/5-FU  Carboplatin/Paclitaxel  Carboplatin monotherapy  Mitomycin C/5-FU  Mitomycin monotherapy  Docetaxel  Cetuximab  Other (please specify) | Single choice  *incl. free-text field* | 67 (78.8) | 18 |
| 17 | What glomerular filtration rate (GFR) or creatinine clearance do you consider as a cut-off for cisplatin eligibility in concurrent chemotherapy? | >60 mL/min  >50 mL/min  >40 mL/min  >30 mL/min | Single choice | 65 (76.5) | 20 |
| 18 | When deciding about concurrent chemotherapy, do you consider hearing loss a cisplatin contraindication? | Yes, ≥ grade 1 (threshold shift of 15-25dB on two contiguous frequencies)  Yes, ≥ grade 2 (threshold shift of >25dB on two contiguous frequencies or hearing aid)  Yes, ≥ grade 3 (threshold shift of >25dB on three contiguous frequencies)  No | Single choice | 68 (80.0) | 17 |
| **Supportive care (items 19–23)** | | | | | |
| 19 | Which supportive measures are routinely provided during the curative treatment of elderly patients at your institution? (Multiple answers possible) | Central venous access  Prophylactic gastrostomy tube placement (PEG)  Speech therapy  Physical therapy  Nutritional counseling  Skin care consultation  Social work services  Other (please specify) | Multiple response  *incl. free-text field* | 66 (77.6) | 19 |
| 20 | Do you routinely offer prehabilitation to elderly patients prior to the initiation of (chemo)radiotherapy? | No  For definitive therapy  For adjuvant therapy preoperatively  Individually (please specify) | Single choice  *incl. free-text field* | 64 (75.3) | 21 |
| 21 | How are elderly head and neck cancer patients typically managed during radiation therapy? | Complete inpatient treatment  Outpatient if medically feasible  Outpatient with inpatient admission for chemotherapy | Single choice | 66 (77.6) | 19 |
| 22 | At what interval are outpatient elderly HNSCC cancer patients seen by a physician during radiation therapy? | Daily  2–3 times per week  Weekly  As needed | Single choice | 66 (77.6) | 19 |
| 23 | How does the follow-up schedule for elderly patients compare to younger patients? | More frequent  Same  Less frequent | Single choice | 66 (77.6) | 19 |
| **Clinical vignettes (items 24–30)** | | | | | |
| *Case 1 (definitive): 76-year-old patient with locally advanced, well-lateralized hypopharyngeal carcinoma cT3 cN0 cM0, non-keratinizing squamous cell carcinoma G3, p16 negative. Staging with panendoscopy and CT of neck, thorax, and abdomen. Karnofsky Index 80%, mild dysphagia and pain. Adequate blood values unremarkable (normal renal function, adequate CBC). Primary definitive therapy planned (surgery declined by the patient).* | | | | | |
| 24 | What treatment strategy would you recommend in this case? | Primary chemoradiotherapy with bilateral cervical nodal irradiation  Primary radiotherapy with bilateral cervical nodal irradiation  Primary chemoradiotherapy with ipsilateral nodal irradiation  Primary radiotherapy with ipsilateral nodal irradiation  Primary chemoradiotherapy of the primary tumor only  Primary radiotherapy of the primary tumor only | Single choice | 63 (74.1) | 22 |
| 25 | What dose prescription would you recommend for the primary tumor region? | 70 Gy (EQD2)  66 Gy (EQD2)  60 Gy (EQD2)  < 60 Gy (EQD2) | Single choice | 63 (74.1) | 22 |
| 26 | What fractionation concept would you recommend in this case? | Conventional (normofractionated)  Hypofractionated  Hyperfractionated  Accelerated | Single choice | 63 (74.1) | 22 |
| *Case 2 (adjuvant): 74-year-old patient with locoregionally advanced oropharyngeal carcinoma (tonsillar region), pT2 pN1 (1/13 nodes with extracapsular spread), cM0. Staging with CT thorax and abdomen. Squamous cell carcinoma G3, p16 positive. Underwent transoral resection and ipsilateral neck dissection with R0 resection. Karnofsky Index 70%.* | | | | | |
| 27 | What treatment strategy would you recommend in this case? | Adjuvant chemoradiotherapy with bilateral cervical nodal irradiation  Adjuvant radiotherapy with bilateral cervical nodal irradiation  Adjuvant chemoradiotherapy with ipsilateral nodal irradiation  Adjuvant radiotherapy with ipsilateral nodal irradiation  Adjuvant chemoradiotherapy of the primary site and involved nodal levels  Adjuvant radiotherapy of the primary site and involved nodal levels  No adjuvant therapy, close imaging follow-up | Single choice | 63 (74.1) | 22 |
| 28 | What dose prescription would you recommend for the primary tumor region? | 63-66 Gy (EQD2)  60 Gy (EQD2)  50 Gy (EQD2)  < 50 Gy (EQD2) | Single choice | 63 (74.1) | 22 |
| 29 | What dose prescription would you recommend for the region of extracapsular spread? | 63-66 Gy (EQD2)  60 Gy (EQD2)  50 Gy (EQD2)  < 50 Gy (EQD2) | Single choice | 63 (74.1) | 22 |
| 30 | What fractionation concept would you recommend in this case? | Conventional (normofractionated)  Hypofractionated  Hyperfractionated  Accelerated | Single choice | 63 (74.1) | 22 |
| **Respondent and institutional characteristics (items 31–34)** | | | | | |
| 31 | At which type of institution do you currently practice? | Academic hospital  General hospital  Outpatient facility | Single choice | 64 (75.3) | 21 |
| 32 | What is your current professional role? | Resident  Board-certified radiation oncologist  Board-certified with more than 10 years of experience | Single choice | 64 (75.3) | 21 |
| 33 | On average, how many elderly head and neck cancer patients are treated at your institution per year? | <10  10-50  51-100  >100 | Single choice | 64 (75.3) | 21 |
| 34 | In what country are you currently practicing? | *Drop-down list of all countries (single selection)* | Single choice | 63 (74.1) | 22 |

*Questions and response options are reproduced verbatim as presented to respondents; the term “elderly” was used in the questionnaire and is replaced by “older” throughout the manuscript. Abbreviations: ADL, activities of daily living; CBC, complete blood count; ECG, electrocardiogram; ECOG, Eastern Cooperative Oncology Group; EQD2, equivalent dose in 2 Gy fractions; GFR, glomerular filtration rate; HNSCC, head and neck squamous cell carcinoma; HPV, human papillomavirus; NRS, Nutritional Risk Screening; PEG, percutaneous endoscopic gastrostomy; SOP, standard operating procedure; WHO, World Health Organization.*

**Supplementary Table 10.** Geographic distribution of respondents by country

*Country of practice was provided by 63 of 85 respondents (74.1%); 22 respondents did not reach the final section of the questionnaire.*

| **Country** | **Respondents, n** | **% of respondents reporting country (n = 63)** |
| --- | --- | --- |
| **Europe (n = 37, 58.7%)** | | |
| *Western Europe (n = 17, 45.9% of European respondents)* | | |
| Germany | 6 | 9.5 |
| Austria | 4 | 6.3 |
| Belgium | 3 | 4.8 |
| France | 2 | 3.2 |
| Netherlands | 2 | 3.2 |
| *Northern Europe (n = 7, 18.9% of European respondents)* | | |
| United Kingdom | 4 | 6.3 |
| Denmark | 2 | 3.2 |
| Ireland | 1 | 1.6 |
| *Eastern Europe (n = 7, 18.9% of European respondents)* | | |
| Poland | 2 | 3.2 |
| Romania | 2 | 3.2 |
| Slovakia | 2 | 3.2 |
| Czechia | 1 | 1.6 |
| *Southern Europe (n = 6, 16.2% of European respondents)* | | |
| Italy | 4 | 6.3 |
| Portugal | 1 | 1.6 |
| Serbia | 1 | 1.6 |
| **Asia (n = 20, 31.7%)** | | |
| India | 9 | 14.3 |
| Türkiye | 3 | 4.8 |
| Bangladesh | 2 | 3.2 |
| Malaysia | 1 | 1.6 |
| Nepal | 1 | 1.6 |
| Singapore | 1 | 1.6 |
| South Korea | 1 | 1.6 |
| Thailand | 1 | 1.6 |
| Vietnam | 1 | 1.6 |
| **North America (n = 4, 6.3%)** | | |
| Canada | 2 | 3.2 |
| Mexico | 1 | 1.6 |
| United States of America | 1 | 1.6 |
| **Africa (n = 1, 1.6%)** | | |
| Tunisia | 1 | 1.6 |
| **Australia (n = 1, 1.6%)** | | |
| Australia | 1 | 1.6 |
| **Total** | **63** | **100.0** |

*Countries are grouped by continent; European sub-regions and the assignment of Türkiye to Asia follow the United Nations M49 geoscheme.*
